# Immuno-psychiatry: an agenda for clinical practice and innovative research

**DOI:** 10.1186/s12916-016-0712-5

**Published:** 2016-10-28

**Authors:** Marion Leboyer, Michael Berk, Robert H. Yolken, Ryad Tamouza, David Kupfer, Laurent Groc

**Affiliations:** 1 Psychiatry Department, University Paris-Est-Créteil, Mondor hospital, AP-HP, DHU PePSY, Translational Psychiatry laboratory, INSERM U955, Paris, France; 2 Fondation FondaMental, Creteil, France; 3 University of Bordeaux, UMR 5297, 33000 Bordeaux, France; 4 Deakin University, IMPACT Strategic Research Centre (Barwon Health), School of Medicine, Geelong, VIC Australia; 5 The Florey Institute of Neuroscience and Mental Health, Orygen, National Centre of Excellence for Youth Mental Health, Parkville, VIC Australia; 6 Department of Psychiatry, University of Melbourne, Royal Melbourne Hospital, Parkville, VIC Australia; 7 Stanley Division of Developmental Neurovirology, The Johns Hopkins University School of Medicine, Baltimore, USA; 8 INSERM, U1160, Hôpital Saint Louis, Paris, F75010 France; 9 Laboratoire Jean Dausset, LabexTransplantex, Hôpital Saint Louis, Paris, F75010 France; 10 Departments of Psychiatry, Western Psychiatric Institute and Clinic, University of Pittsburgh School of Medicine, Pittsburgh, PA USA; 11 Interdisciplinary Institute for Neuroscience, CNRS UMR 5297, 33077 Bordeaux, France

**Keywords:** Personalised psychiatry, Immune inflammation, Biomarkers, Biological pathways, Translational research

## Abstract

**Background:**

The diagnostic scheme for psychiatric disorders is currently based purely on descriptive nomenclature given that biomarkers subtypes and clearly defined causal mechanisms are lacking for the vast majority of disorders. The emerging field of “immuno-psychiatry” has the potential to widen the exploration of a mechanism-based nosology, possibly leading to the discovery of more effective personalised treatment strategies.

**Discussion:**

Disturbances in immuno-inflammatory and related systems have been implicated in the aetiology, pathophysiology, phenomenology and comorbidity of several psychiatric disorders, including major mood disorders and schizophrenia. A fundamental challenge in their clinical management is to identify bio-signatures that might indicate risk, state, trait, prognosis or theragnosis. Here, we provide the rationale for a clinical and research agenda to refine future clinical practice and conceptual views, and to delineate pathways toward innovative treatment discovery.

**Conclusion:**

The development of bio-signatures will allow clinicians to tailor interventions to the abovementioned biomarker subtypes – a major translational goal for research in this field.

## Background

Despite increasing scientific knowledge and enormous efforts to classify psychiatric disorders, this field is currently limited by the absence of an articulated neurobiological substrate to delineate valid diagnostic entities, inhibiting the development of timely, explanatory, mechanism-based therapeutic strategies. Further, this constitutes a major driver for the failure to discover novel therapies for those with neuropsychiatric disorders [1]. Current diagnostic schemes for psychiatric disorders are based on descriptive nomenclature given the absence of clearly defined causal mechanisms. Such descriptive nomenclature does not easily include the multiple and successively occurring psychiatric and medical comorbidities, nor the considerable diagnostic instability demonstrated in longitudinal studies.

Disturbances in the immuno-inflammatory system have been implicated in the aetiology, pathophysiology, phenomenology and comorbidity of several psychiatric disorders such as schizophrenia, bipolar disorder, major mood disorders, suicidal behaviour, post-traumatic disorder and autism [2, 3]. Inflammation and activated cell-mediated immune pathways have consistently been found in diverse patient cohorts indexed by an up-regulated expression of pro-inflammatory cytokines [4, 5], triggered and sustained by environmental risk factors such as very early infections [6], severe childhood trauma [7] or lifelong psychosocial stressors [8]. It is noteworthy that inflammatory biomarkers overlap with risk pathways for medical disorders commonly comorbid with psychiatric disorders such as diabetes, cardiovascular disease and osteoporosis [9]. In this context, the emerging field of “immuno-psychiatry” has the potential to widen the exploration of a mechanism-based nosology, possibly leading to the discovery of more effective personalised treatment strategies. Herein, we provide the rationale for a research agenda to refine clinical practice, including the description of steps to define the basis of an immuno-psychiatry-based nosology, as well as pathways toward innovative treatment discovery. We describe putative research areas and delineate, for each of them, the current evidence, associated obstacles and research strategies to inform treatment decisions and to build an agenda for treatment discovery.

Diagnosis in psychiatry is still restricted to symptoms and observable signs given the lack of valid biomarkers. Most biomarker candidates show insufficient sensitivity and specificity within disorders and overlap greatly between disorders [10]. Furthermore, diagnostic classification does not consider the medical and psychiatric comorbidities nor the lifetime staging processes [11]. Therefore, efforts to reformulate DSM-5 and ICD-11 nosology should be developed through careful exploration of possible mechanisms (e.g. dimensions, categories, environmental risk factors and/or biological markers) and should lead to, and ultimately be validated by, insightful and targeted therapeutic strategies. Along the same line, primary prevention strategies based on the identification of modifiable environmental risk factors associated with inflammation need to be conceptualised [2]. It is known that many of the known risk factors for disorders are indeed transduced via inflammatory and related pathways such as redox biology [2]. Indeed, we would argue for a common framework of conceptualised psychiatric disorders among the disproportionally comorbid non-communicable medical disorders because of shared antecedent risks and common biomarkers. This framework suggests and, as we will later illustrate, shows evidence for common treatment and preventive strategies. Linking cross-clinical dimensional symptomatology with epidemiological and biological observations may help refine meaningful new subgroups using environmental risk factors, such as infections or stress, to assess medical comorbid disorders and immune phenotypes to inform treatment.

## Auto-immunity, early infection and immune-genetic background: characterisation for prevention?

Autoimmune diseases and early infections are well-known to increase the risk of subsequent psychiatric disorders, including mood disorders, autism spectrum disorders or schizophrenia [3, 12, 13]. For instance, a history of hospitalisation for infection is known to increase the risk of later mood disorders by 45 %, while prior history of auto-immune disorders and infections interact in synergy to further raise the risk of subsequent mood disorders by more than 60 % [14]. Maternal history of auto-immune disorders is also associated with an increased risk of subsequent autism spectrum disorders in offspring of more than 40 % [15], whereas maternal infections requiring hospitalisation have been reported in autism spectrum disorders [16]. Auto-immunity in schizophrenia, affective psychosis and bipolar disorder has also been reported [17], thus clearly crossing classical diagnostic boundaries [6]. Cognitive decline and brain imaging abnormalities have also been associated with prenatal infections and/or elevated cytokine levels across disorders [18].

How to translate these findings into an agenda? Not all subjects that experience early life infection develop psychiatric disorders. Environmental factors, such as early infection, could predispose to autoimmune disturbances in immunogenetically vulnerable individuals. A relevant validation strategy would thus be to search for associations between immunogenetic variants and life infection history. In animal models, for instance, transgenic mice displaying a genetic predisposition to autism exhibit enhanced responses to environmental challenges to maternal immune activation [19]. In humans, the major histocompatibility complex region that includes the human leukocyte (HLA) genes locus, which has a crucial role in initiating cellular and humoral immune responses as well as vulnerability to autoimmune disorders, appears as a major candidate to identify at-risk subjects. Several HLA genes and haplotypes are linked to autism [13], while HLA polymorphisms are amongst the most associated region in schizophrenia [20]. Furthermore, an association between bipolar patients with autoimmune comorbid disorders and genes encoding functional Toll Like Receptors (innate immunity) has been reported [21]. Strengthening the exploration of immunogenetic diversity in major psychiatric disorders, such as innate, adaptive immunity and inflammation pathways, is urgently needed as has been performed for cancer [22]. It is necessary to further test gene–gene interactions between risk pathways and the immune and synaptic pathways as demonstrated in autism [23] and schizophrenia [24]. This will permit identification of at-risk individuals and their follow-up along the developmental exposure to various infections and other environmental risk factors, additionally suggesting developmental pathways for novel therapeutic strategies.

## Developmental stress: another factor for resilience/susceptibility to mental disorders?

Besides the impact of early infections/autoimmune disorders in genetically sensitive subjects, a second “hit”, such as postnatal stress, also predisposes these individuals to physiological abnormalities mediated by prenatal immune activation. Indeed, early exposure to trauma in childhood can increase the subsequent risk of poor functioning of the immune and endocrine systems, which could constitute risk factors for later depression [7]. Early life stress in humans has been implicated in many psychiatric disorders in adult life, ranging from depression to schizophrenia, as well as somatic illnesses ranging from cardiovascular illness and diabetes to alterations at all levels of the brain–gut axis such as the altered balance of enteric microflora [25]. Early life stress is also known to induce long-term alterations in inflammatory responses, including elevated C-reactive-protein and pro-inflammatory cytokines (IL-6, TNF-α, Th1-like) [26]. Bipolar patients with a history of childhood trauma have a more severe form of illness characterised by earlier age at onset, suicide attempts, rapid cycling, an increased number of depressive episodes, and higher levels of inflammatory markers [27]. Adult vulnerability to neuropsychiatric diseases can thus be conceived as being imprinted during foetal and early postnatal periods through genetic and/or epigenetic mechanisms [28]. It must be noted at this juncture that immune regulatory pathways do not operate singularly, but form part of a complex interacting network with other pathways, including, but not limited to, redox, neurogenesis/apoptosis, mitochondrial function and other stress response pathways [29].

How to translate these findings into an agenda? A subgroup defined by history of severe and early stress could be identified by psychological and biological signatures associating, for instance, elevated levels of cytokines, cortisol and oxidative stress with specific clinical characteristics. These could be further refined by developmental animal models in which the phenotypic target of the pre/postnatal stress model, e.g. specific molecular, behavioural or cognitive deficit [30, 31], higher susceptibility to environmental challenges, or altered impact on risk and vulnerability pathways [32], will be defined. Life stressors are likely to occur at different periods, with different origins and intensities, and their impact could also be tested in these animal models. Furthermore, developmental stress implicates biological mechanisms in which numerous bodily systems, both peripheral and central, are engaged. Monitoring these parameters in classical pre/postnatal stress models and testing the resistance to stress after manipulations of these parameters will shed new light on how environmental challenges early in life may or may not alter resilience and vulnerability pathways. Finally, at-risk subjects exposed to early and/or chronic stressors could also be identified by their immunogenetic markers as known factors for higher risk of psychiatric disorders (see above). Collectively, we advocate herein that early identification of “high at-risk” subjects who underwent severe stress events during childhood will thus open the possibility of prevention strategies based on changeable environmental risk factors and restored balance in key biological systems.

## Medical comorbidities in major psychiatric disorders: more than a coincidence?

Comorbidity between psychiatric and medical disorders is a major issue as it is estimated that approximately 70–90 % of individuals with bipolar disorder experience a concomitant psychiatric or somatic disorder [33]. Compared to the general population, metabolic syndrome is twice more common in patients with bipolar disorder and schizophrenia [34]. Further, an array of disorders is more commonly comorbid with depression (e.g. atopy, cardiovascular disorders, osteoporosis and diabetes). Unsurprisingly, they all share an inflammatory component to their aetiology. Although these comorbid patterns have long been viewed as consequential to an unhealthy lifestyle or to the side effects of psychotropic drugs, it has been demonstrated that a pro-inflammatory signature, with elevated IL-1, IL-6, cortisol and leptin levels along with obesity, was observed in subjects already at risk of depressive, bipolar or psychotic disorders [35–37]. Similarly, the large majority of young subjects with autism spectrum disorders exhibit comorbid patterns, with more than 90 % suffering of gastrointestinal problems [38]. Abnormal metabolic and inflammatory signatures are thus associated with higher susceptibility for both comorbid medical and mental disorders [9].

**Fig. 1:**
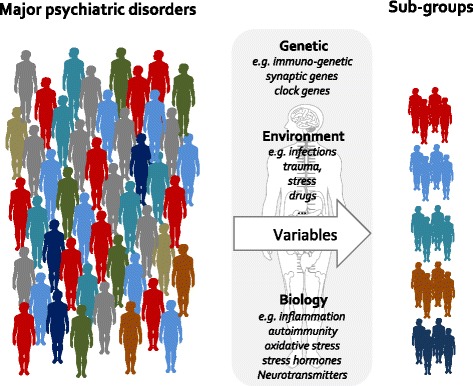
Schematic illustration of the putative diversity within a psychiatric patient cohort (e.g. schizophrenic ones) diagnosed with a descriptive, and not mechanistic, nomenclature. The emerging field of immuno-psychiatry has the potential to open up the exploration of mechanism-based nosology and personalised treatment strategies once multiple variables (e.g. genetic, environmental, biological) are integrated to identify patient subgroups

How to translate this observation into an agenda? Available data strongly suggests that currently defined psychiatric disorders could be either (or simultaneously) composed of diverse biological entities fitting under a same phenomenological spectrum umbrella or different phenomenological variants of a common underpinning pathophysiological process. First, we could use advanced methods, such as systems biology and theoretical “big data” bioinformatics, to identify new clusters of patients, integrating the complex phenotypic, anamnestic, psychiatric and somatic comorbidities, as well as familial and environmental risk factors, longitudinal history and biomarker profiling to shed light on these heterogeneous disorders [39]. The oncology field has successfully applied these techniques to evaluate the relevance of groups of factors and to identify bio-based clusters amenable to personalised treatment. Lifespan human and animal models that take into account the successive impact of environmental factors are also warranted to better understand the interactive and/or additive effect of these factors on the course of psychiatric disorders. Ideally, biological markers already identified in these disorders or extracted from the above emerging models in derivation studies could serve to design new “educated chips” that will contain immune, neurobiological and genetic markers defining subtypes of patients that could be further refined in validation studies (Fig. 1).

The use of animal models could facilitate exploration of in-depth temporal and molecular pathways linking central and peripheral dysfunctions in psychiatric disorders. For instance, since the discovery that some NMDA receptor (NMDAR) blockers induce schizophrenia-like psychosis, reproducing both positive and negative symptoms, the NMDA glutamatergic model of psychosis and schizophrenia has been increasingly accepted as part of the disease’s etiopathology [40]. The dysfunction of NMDAR signalling might originate, among other pathways, from genetic alteration, autoantibodies directed against extracellular epitopes [41–46], and/or altered levels of endogenous agonists/antagonists (kynurenine metabolites) [47]. Of note, the recent discovery that autoantibodies against extracellular epitopes of the NMDAR produce major psychosis [41–46] provides a unique opportunity to increase our understanding of psychotic disorders at the molecular, cellular and brain imaging levels. For instance, autoantibodies from anti-NMDA encephalitis patients altered NMDAR signalling by preventing molecular interaction and altering membrane trafficking of NMDAR, although without modulating the function of the NMDAR channel [43]. Thus, NMDAR dysfunction-induced psychosis could originate from a blockade of the channel (e.g. phencyclidine) or an altered trafficking of the receptor (e.g. autoantibodies). Future investigations in various psychotic disorders will thus shed new light on the mechanisms underlying these disorders and pave the way for new, innovative therapeutical strategies to restore proper NMDAR signaling.

**Fig. 2:**
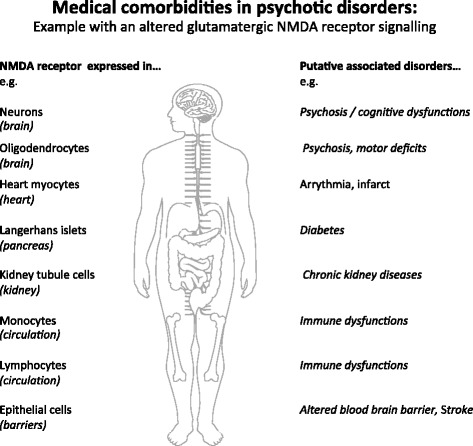
Example of the putative molecular link between medical comorbidities in psychotic disorders. An alteration of the glutamatergic NMDA receptor signalling, proposed to be central in the aetiology of psychotic disorders, may have varying impact in the various organs of these patients

Could a common dysfunction be at the origin of peripheral disorders? Theoretically, yes. Indeed, it is often forgotten that NMDAR is not only expressed in brain cells but also in many other organs and cell types; for example, NMDAR is expressed in pancreatic islets and in insulin-secreting beta cells whose functional impairment contributes to diabetes. A recent study demonstrated that inhibition of NMDAR in mouse and human islet cells enhanced their glucose-stimulated insulin secretion and survival [48]. Thus, alterations in NMDAR signalling might directly confer risk for psychosis and diabetes. In addition, NMDAR is also expressed at the surface of epithelial barrier cells [49], monocytes [50] and lymphocytes [51], potentially regulating biological barriers and immune reactions. Finally, NMDAR is expressed by cardiac myocytes and cells of the basolateral proximal tubule in the kidney, and its activation in these systems produces important cellular and organ regulation (see [52] for a review). Thus, in addition to its well-established role in the aetiology of major psychiatric disorders, alterations of NMDAR signalling could also sustain comorbid disorders in these patients (Fig. 2). Innovative and longitudinal models urgently need to be implemented in order to investigate the shared molecular pathways that could link comorbid somatic and psychiatric disorders as a whole.

## Agenda for innovative treatment: new ways to prescribe existing drugs?

Despite major advances in the understanding of severe psychiatric disorders, the number of new drugs made available is steadily declining [53]. Improving our understanding of the biological networks and systems associated with these complex disorders as well as identifying mechanism-based subgroups among large and complex psychiatric entities would facilitate a shift towards target-based high-throughput screening of large compound libraries as advocated for drug discovery [1]. If it is confirmed that subgroups of patients are characterised by factors such as infections, autoimmunity, inflammation and immunogenetic-mediated response to environmental factors, then several existing agents could be prescribed on the basis of their actions on the immune system [54]. For example, toxoplasmosis is the most common protozoa parasite infecting people with psychiatric disorders, with a 2.7-fold increase in overall odds of *T. gondii* seropositivity [55], as recently confirmed by a meta-analysis showing an association with *T. gondii* antibodies (immunoglobulin type G) in schizophrenia and obsessive–compulsive disorder [56]. Remarkably, bipolar disorder and schizophrenic patients seropositive for *T. gondii* appear to have a better outcome when receiving antipsychotics with known in vitro anti-toxoplasmic activity when compared to patients receiving a treatment without anti-toxoplasmic activity [57].

One may also repurpose drugs that directly target inflammatory pathways to subgroups of patients with immune phenotypes. For instance, the tetracycline antibiotic minocycline, which exhibits anti-inflammatory, pro-oxidant, glutamatergic and neurotrophic features, has been successfully tested in schizophrenia and major depressive disorders [58, 59]. Both aspirin [60] and non-steroidal anti-inflammatory drugs (NSAIDs) [59] reduce positive and negative symptoms of schizophrenia when used as add-ons to standard antipsychotic therapy, whereas add-on celecoxib has anti-depressant effects [61]; the efficacy of NSAID therapy is now supported by meta-analysis [62]. N-acetylcysteine, which has redox, glutamatergic and anti-inflammatory properties, diminished symptom severity in add-on studies in schizophrenia and showed antidepressant effects in bipolar disorder and depression [63]. Ketamine and its metabolites worked in depression via their glutamatergic properties [64] as well as through robust effects on microglial suppression [65]. It is noteworthy that high inflammation has been shown to impair, to a certain extent, the action of antidepressants and antipsychotics [66, 67]. Thus, subgroups of psychiatric patients with infectious and inflammatory conditions should strongly benefit from adapted therapies, either with direct anti-inflammatory drugs or with antipsychotics with inherent anti-inflammatory properties.

### New treatments based on newly-validated targets

The discovery and development of new medications for clinically and immunologically stratified subgroups of patients would represent a major breakthrough in the field of psychiatric disorders. New targets for pathway-driven nosology will be as important for the future as the above refined strategies. One may envision that developing new modulators of specific cytokines (e.g. neutralising interleukins), products of infection-activated human endogenous retrovirus (e.g. HERV-W), or peripheral and central nervous system receptors (e.g. glutamate NMDAR subunits) would be of great interest for the subgroup of patients with these immune phenotypes. For instance, biological immunotherapy that directly targets cytokines such as IL-6 are worthy of interest since elevated levels of IL-6 have been reported in and may serve as markers of both schizophrenia and mood disorders [68]. As antibodies opposing the effects of IL-6 have been successfully developed for autoimmune diseases, they are promising candidates for psychiatric disorders, although probably only in the presence of immune abnormalities such as elevated levels of IL-6, autoantibodies and infectious stigma. Another targeted immunotherapy might be the use of antibodies neutralising the envelope protein of the human endogenous retrovirus HERV-W, found elevated in both bipolar disorder and schizophrenia [69], since the neutralising antibody GNbAC1 abrogates the HERV-W envelope protein and has been successfully tested in clinical trials of patients with multiple sclerosis [70].

Nevertheless, the immense efforts made over the past decades, in particular by major pharmaceutical companies, to target the neurotransmitter receptor signalling pathways extensively correlated to psychiatric disorders have not paid off. Two new lines of exploration could be envisioned. First, most efforts have concentrated on the development of drugs with a direct modulatory effect on the receptor, whereas an emerging array of evidence point toward a major defect of neurotransmitter receptor trafficking in brain cells. Focusing on innovative strategies to restore proper trafficking, and thus signalling, constitutes a promising research area. Second, the emerging functional interplay between synaptic and immune receptors, supposedly involved in brain connection maturation during development [71] and psychiatric disorders [72], should prompt us to build mechanistic and integrated models to leverage the immense potential of the genome-wide association studies highlighting the interconnection between neurotransmission and immunity in psychiatric disorders.

## Conclusion

Beyond the enormous economic burden that psychiatric disorders represent, the discovery of alterations in the intimate molecular networks that sustain the diseases and their application to develop novel therapeutic strategies is one of the most exciting and key scientific challenges for the 21st century. Here, we advocate that the path to discovery would first require a “deconstruction” of current practices to segment psychiatric disorders and we suggest elements of a research agenda to adapt clinical practice and foster research paradigms. The grounds for reconstruction should be based on the integration of a reconciled body–brain interface leading to a new “immuno-psychiatry-based nosology”. Indeed, the accumulating evidence that immune and environmental alterations play a key role in the aetiology of seemingly diverse disorders provides a promising opportunity to define latent subgroups that share immune, genetic and brain alterations. Innovative animal models are within our grasp to fully identify and properly address emerging obstacles. The opportunities for therapeutic strategies, both by re-purposing existing drugs and by identifying targets with new angles, may be immense and could provide the necessary hope that clinicians, pharmaceutical companies and, most importantly, patients may have gradually lost over the past decades.
